# Trajectories of Job Burnout among Bus Drivers in China: A Three-Year Follow-Up Study

**DOI:** 10.3390/ijerph192417098

**Published:** 2022-12-19

**Authors:** Andi Huang, Lili Liu, Xiayong Wang, Xueguo Li, Jiahong Li, Cong Luo, Jianbin Chen, Jingbo Zhao

**Affiliations:** 1 Department of Psychology, School of Public Health, Southern Medical University, Guangzhou 510515, China; 2 Mental Health Education and Counseling Center, School of Public Health, Southern Medical University, Guangzhou 510515, China

**Keywords:** burnout, drivers, longitudinal, multinomial logistic regression, trajectories, growth mixture modeling

## Abstract

This study aimed to characterize job burnout in longitudinal trajectories among bus drivers and examine the impact of variables related to job burnout for trajectories. A longitudinal study was conducted in 12,793 bus drivers in Guangdong province, China, at 3-year follow-up assessments. Growth mixture modeling (GMM) was used to estimate latent classes of burnout trajectories and multinomial logistic regression models were applied to predict membership in the trajectory classes. In general, there was a decrease in job burnout in 3 years [slope = −0.29, 95%CI = (−0.32, −0.27)]. Among those sub-dimensions, reduced personal accomplishment accounted for the largest proportion. GMM analysis identified five trajectory groups: (1) moderate-decreased (*n* = 2870, 23%), (2) low-stable (*n* = 5062, 39%), (3) rapid-decreased (*n* = 141, 1%), (4) moderate-increased (*n* = 1504, 12%), and (5) high-stable (*n* = 3216, 25%). Multinomial logistic regression estimates showed that depression symptoms, anxiety symptoms, and insomnia were significant negative predictors, while daily physical exercise was a significantly positive predictor. We found an overall downward trend in bus drivers’ burnout, particularly in the sub-dimension of personal accomplishment. Mentally healthier drivers and those who were usually exercising were more resilient to occupational stress and less likely to suffer burnout.

## 1. Introduction

Stress at work is an increasingly common phenomenon in society. Over a period of time, this stress may result in exhaustion, named burnout syndrome, which has been recognized as an occupational health problem [1]. Previous study has showed that the prevalence of work-related stress and burnout was significantly high among bus drivers [2]. Bus drivers are prone to job burnout because of various complicated trivial problems of passengers and work in fixed positions for a long time [3,4]. However, there have been few research studies conducted to characterize burnout in professional bus drivers.

Burnout is a long-term response to chronic emotional and interpersonal stress at work and is defined by three dimensions: exhaustion, cynicism, and inefficacy [5]. The dimension of exhaustion was represented as weariness, loss of energy, depletion, weakness, and fatigue. Cynicism, originally called depersonalization, was described as negative or inappropriate attitudes towards clients, irritability, loss of idealism, and withdrawal. Inefficacy, previously named reduced personal accomplishment, was described as reduced productivity or capability, low morale, and an inability to cope [5].

There are various theory models of the formation and development of burnout. Some models focus on the relationship between the three dimensions of burnout, which are usually described as successive stages [5]. Exhaustion first arises in situations of high demand and overload, and this then leads to detachment and negative reactions to people and work (depersonalization or cynicism). If this persists, then the next stage is a feeling of inadequacy and failure (reduced personal achievement or professional inefficiency). Previous studies have suggested that burnout is a gradual process [6,7]. For example, in a 2-year follow-up study in nurses, physicians, managers, and administrators, Benjamin B. D. found that burnout was relatively stable for employees without company changes [8].

In addition, there are theories that focus on resources. For example, the Job Demands-Resources (JD-R) model suggests that when an individual’s job demands are increasing, but there are not enough resources to meet and cope with these demands, this is when the burnout happens [9]. Bus drivers are a special profession. They constantly repeat the same route, repeat the same work, and are required to spend long hours in the driver’s seat and be flexible and responsive to the needs of passengers. They put a lot of effort into their work and sometimes find it difficult to receive positive feedback [10]. In China, drivers often work upwards of 10 h each day, with 12–14 h of work being the norm, and often have less than half an hour of rest at the final station each time [11]. With longer work hours and less social and family time, drivers have fewer opportunities to receive social and family support, making them more likely to suffer from burnout [10].

Previous research has found gender [12,13], age [12,13,14], and social environmental factors (e.g., marriage status [15], number of children [16,17]) were associated with burnout. Work-related factors, such as job stress [18,19], effort–reward imbalance [20], low social support [4,16,21], and working time [13,15] were associated with burnout. Especially for the driver group, length of driving years [22] and working hours [14] were also to be considered [23]. Job burnout was significantly correlated with depression [16,24,25], anxiety [24], stress [26], and insomnia [27,28].

In a study of professional drivers in China, job burnout was positively associated with aggression, which may be related to long working hours, poor working conditions, lack of discipline, and even passenger verbal and physical aggression [29]. Although the negative outcome of job burnout has been confirmed in various occupations, little is known about trajectories of burnout and their predictive factors among Chinese bus drivers. In summary, we hypothesized that demographic information (age, gender, marital status, number of children), work-related factors (financial situation, body disease, driving years, daily work hours, daily physical exercise), and mental health factors (mental illness, depression, anxiety, insomnia, aggression) may be predictive factors for trajectories of burnout in bus drivers.

The aim of this study was to identify and characterize potential job burnout trajectories and the relationship between job burnout and other related variables. By using the growth mixture modeling (GMM), we were able to offer a long-term understanding of job burnout for bus drivers and to provide evidence to develop better occupational mental health strategies.

## 2. Materials and Methods

### 2.1. Designs and Participants

The study was a repeated measurement study performed at three time points. The three examined times were T1: 10 August to 9 October 2019; T2: 9 October to 9 December 2020; T3: 21 October to 9 December 2021. Bus drivers in Guangdong province, China, were invited to participate through online questionnaires using Sojump (for more information about Sojump, see Supplementary Material File S3). The number of participants was 30,351, 23,116, and 19,855 in T1, T2, and T3. In total, 17,080 participants finished all three surveys, where 12,793 (valid questionnaire: 74.9%) valid surveys were collected.

### 2.2. Materials

#### 2.2.1. Demographic

Demographic factors, age, gender (male or female), marital status (married, unmarried, divorced, or widowed), the number of children, financial situation (How would you rate your family’s financial situation?), body disease (Are you suffering from severe physical disease?), mental illness (Have you ever been diagnosed with a mental illness?), driving years (How many years have you been in the business?), working hours (How many hours do you work per day?), and daily physical exercise (In the past two weeks, what was the average amount of time spent exercising daily?) were determined. More information can be seen in Supplementary Material File S1.

#### 2.2.2. Job Burnout

The Chinese version of Maslach Burnout Inventory-General Survey (MBI-GS) was used to measured burnout among bus drivers [30]. The MBI-GS questionnaire included 15 items and 3 dimensions. The three dimensions were: Emotional Exhaustion, Cynicism, and Reduced Personal Accomplishment. Emotional Exhaustion included five items, which were used to evaluate stress-induced emotional responses. Cynicism included four items, which were used to evaluate the attitudes and feelings caused by workplace stress. Reduced Personal Accomplishment included six items and was mainly used to assess the personal accomplishment in the workplace. In this dimension, items need to be recorded in reverse. The total score range is from 0 points to 90 points. Each item has 7 options (0 = “never”, 1 = “very rarely (a few times a year or less), 2 = “occasionally (once a month or less)”, 3 = “often (a few times a month)”, 4 = “frequently (once a week)”, 5 = “Very often (several times a week)”, 6 = “daily”). Higher summed scores reflect higher levels on job burnout. The Cronbach’s α were 0.87, 0.86 and 0.83 at T1, T2 and T3, respectively.

#### 2.2.3. Depression Symptoms

The Chinese version of the Patient Health Questionnaire (PHQ-9) was used to measure depression symptoms. PHQ-9 measures depression symptoms within two weeks [31]. The total score is range from 0 points to 27 points with each item having 4 options (0 = “not at all”, 1 = “a few days”, 2 = “more than half the days”, 3 = “nearly every day”) and higher scores reflect the higher levels of depression. The total cutoff score in this investigation was 5 (less than or equal to 4 was coded as 0, and greater than or equal to 5 was coded as 1). The Cronbach’s α of PHQ-9 in this research was 0.91 at T1.

#### 2.2.4. Symptoms of Anxiety

The Chinese Generalized Anxiety Disorder Scale (GAD-7) was used to measure anxiety symptoms [32]. The total score range is from 0 points to 21 points with each item having 4 options (0 = “not at all”, 1 = “a few days”, 2 = “more than half the days”, 3 = “nearly every day”) and the higher scores reflect the higher levels of anxiety. In the current study, the total cutoff score was 5 (less than or equal to 4 was coded as 0, and greater than or equal to 5 was coded as 1). The Cronbach’s α of GAD-7 in the research was 0.94 at T1.

#### 2.2.5. Insomnia

We used the Insomnia Severity Index (ISI) for evaluation of bus drivers’ insomnia [33]. The ISI measures sleep problem within two weeks. The total score range is from 0 points to 28 points and high scores indicated greater insomnia severity. In the current study, the total cutoff score was 7 (less than or equal to 7 was coded as 0, and greater than to 7 was coded as 1). The Cronbach’s α of ISI in the research was 0.92 at T1.

#### 2.2.6. Aggression

The Chinese version of the Buss–Perry Attack Scale was used to measure aggression among drivers [34]. It ranges from 0 points to 28 points, and a higher score suggests more assertive, aggressive behavior. The Cronbach’s α of Buss–Perry Attack Scale in this research was 0.95 at T1.

### 2.3. Statistical Analyses

Latent class growth analyses were analyzed using Mplus, version 8.0. GMM analysis was an iterative procedure. It starts from a one-class model, and then successive models extract additional classes. After analyzing the one-class model, a two-class model was examined, followed by a three-class model, and so on. Model solutions were evaluated according to the Akaike Information Criteria (AIC), Bayesian Information Criterion (BIC), Entropy, posterior class probabilities, and the Adjusted Lo–Mendell–Rubin (LMR) test. Lower BIC and AIC values, as well as higher Log-Likelihood values, indicate a better model fit. The model’s classification accuracy was summarized using entropy, which ranged from 0 to 1 and more than 0.8 meant that the classification quality of the model was acceptable. Based on the best-fit statistics of the model, the model was selected with the optimal number of classes. After selecting the best fit model, predictors of latent class membership were examined.

A multinomial logistic regression analysis was conducted using Mplus 8.0 in order to identify predictors that distinguished the different trajectories, with class trajectories as dependent variables. The prediction variables were as follows: age, gender, marital status, number of children, financial situation, mental illness, driving years, working hours, daily physical exercise, depression symptoms, anxiety, insomnia, and aggression. Among these independent variables, age, the number of children, driving years, and aggression were continuous variables, and all other independent variables were coded as dichotomous variables. The Code of Latent class growth and multinomial logistic regression are visible in Supplementary Material File S2. The figure was done using Graphpad prism.

## 3. Results

### 3.1. Characteristics of Study Subjects

As shown in Table 1, the mean age of these 12,793 bus drivers (98.60% were male) was 47.27 years with an average of 12.39 driving years, where 11,399 participants (89.710%) were married and had 1.49 children on average. Furthermore, 6006 participants (46.95%) felt they were in a lower financial situation, and 53,827 participants (62.0%) were in good mental health. About 57.46% of participants work more than 8 h, and 43.91% do physical exercise for more than 30 min every day. Additionally, 2677 (20.93%), 1808 (14.13%), and 1923 (15.03%) bus drivers had symptoms of depression, anxiety, and insomnia, respectively. The driver’s aggression score was 39.29.

### 3.2. Scores of Job Burnout Were Decreased in Bus Drivers

**Figure 1:**
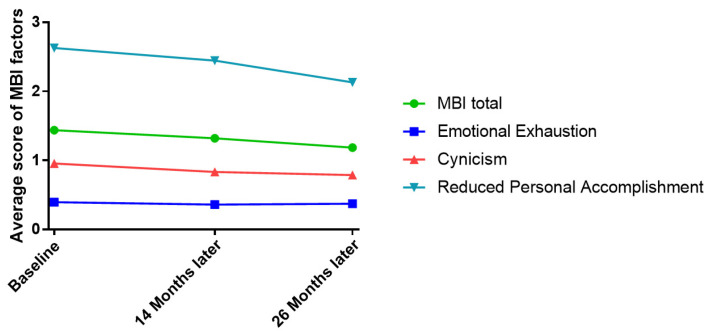
The estimated average score of dimensions of job burnout.

Because each dimension had a different number of items (e.g., emotional exhaustion has five items, cynicism has four items, and reduced personal accomplishment has six items), we used the mean score of each dimension for comparison. As shown in Figure 1, over the 26 months, there was a decrease in MBI total score, emotional exhaustion, cynicism, and reduced personal accomplishment. The highest average score appeared in the dimension of reduced personal accomplishment, while the lowest average score appeared in emotional exhaustion. The slopes varied across total scores and different dimensions. For instance, there was a decline in MBI total score (slope = −0.29, 95%CI = (−0.32, −0.27), *p* < 0.00) and also a decline in reduced personal accomplishment (slope = −0.23, 95%CI = (−0.25, −0.21), *p* < 0.00), but little change was observed in emotional exhaustion (slope = −0.05, 95%CI = (−0.06, −0.04), *p* < 0.00) and in cynicism (slope = −0.01, 95%CI = (−0.02, 0.00), *p* < 0.00).

### 3.3. Selection of the Best-Fitting Model

Table 2 presented the fit indices for 1 to 6 latent class solutions. According to the fit indices, it was concluded that, of the job burnout trajectories the 5-class solution was the best-fitting model (AIC = 308,689, BIC = 308,838). The sample sizes in this model were acceptable (23%, 39%, 1%, 12%, and 25% of the sample). This model included 20 parameters and a high entropy at 0.909 (well above the 0.80 cutoffs), indicating a remarkable homogeneity among groups, and higher entropy and latent class probabilities indicate greater parsimony. The LMR was significant at 1060.

The average latent class probabilities for most likely latent class membership is presented in Table 3, where the 5-class solution was high at 93.2%, 95.1%, 90%, 92.3%, and 94.6%. These results underline the reliability of the 5-class trajectory.

### 3.4. Trajectory Analysis

**Figure 2:**
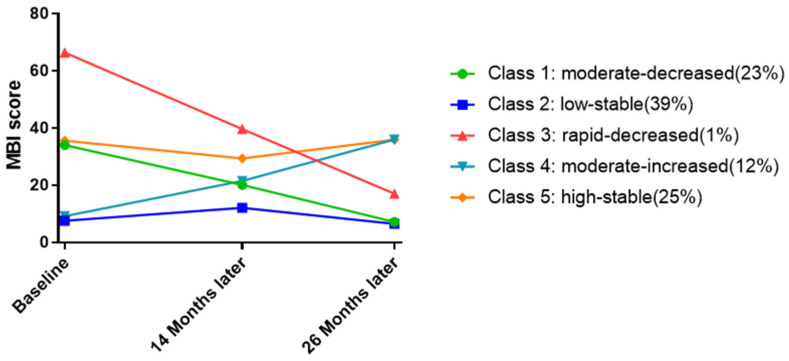
Job burnout trajectories identified by GMM.

Figure 2 displays the estimated means of job burnout for each group identified from the final parsimony model by the GMM analyses, based on observed MBI scores at T1, T2, and T3. Slopes with absolute values less than 1 were classified as stable, while slopes greater than 1 were classified as increased or decreased.

The largest class (class 2, *n* = 5062) was characterized by a low slope (slope = −0.08, *p* < 0.00) and a low intercept (intercept = 8.20, *p* < 0.00) compared to others. Though the slope in class 2 was significant, the absolute value was close to 0. Therefore, class 2 was termed “low-stable”. The same as “low-stable”, class 5 (*n* = 3216) had a stable slope (slope = 0.02, *p* = 0.13) while class 5 had a higher intercept (intercept = 35.09, *p* < 0.00) compared to “low-stable”. Therefore, class 5 was termed “high-stable”. The third largest class (class 1, *n* = 2870) also had a high intercept (intercept = 34.22, *p* < 0.00) and a stable slope (slope = −2.07, *p* < 0.00), so we named it “moderate-decreased”. Compared to “moderate-decreased”, class 3 (*n* = 141) had a more oblique slope (slope = −3.80, *p* < 0.00) and had the highest intercept (intercept = 66.49, *p* < 0.00), so class 1 was termed “rapid- decreased”. The last one was class 4 (*n* = 1504) and was characterized by an increased slope (intercept = 9.08, *p* < 0.00), therefore termed “moderate-increased”.

Therefore, the five groups were named separately: (1) the moderate-decreased, (2) the low-stable, (3) the rapid-decreased, (4) the moderate-increased, and (5) the high-stable.

### 3.5. Burnout-Related Predictors of Functioning Trajectories

Results of the multinomial logistic regression were shown in Table 4. Job burnout-related variables were examined as potential predictors of the different trajectories. Different trajectories were fixed as the reference class in response to our most curious observations.

“Moderate-increased” and “low-stable” had comparable scores of MBI at T1, but one score became higher, and one score remained stable. We were curious about the potential predictors for this, so we set “low-stable” as the reference class. Similarly, we also set “moderate-decreased” as the reference class to find the potential factors that “moderate-decreased” and “high-stable” trends were different. “Low-stable” and “high-stable” were both stable, but we were curious as to why one scored high and one scored low, so we set “low-stable” as the reference class. In the two trajectories, “moderate-increased” and “moderate-decreased”, one was increased and one decreased. We were also curious about what their underlying factors were, so we set “moderate-decreased” as the reference class.

The overall results for the multinomial logistic regression showed that daily physical exercise, depression symptoms, anxiety symptoms, and insomnia were significant predictors. Daily physical exercise for more than 30 min was a protective factor, while depression, anxiety, and insomnia were risk factors.

In addition to these factors (daily physical exercise, depression symptoms, anxiety symptoms, and insomnia), older drivers or those with more years of driving experience were more likely to have a moderate increase in MBI than those who had been stable-low. Drivers with lower than normal economic status or higher aggressiveness were more likely to be on a stable-high trajectory than a moderate-decreased trajectory. Lower financial situation, aggression, and longer driving years predicted a high stable trajectory in contrast to a stable low trajectory, while number of children predicted a stable low trajectory in contrast to a high stable trajectory. Older, more children, lower financial situation, and longer daily working time predicted a moderate increase in MBI compared to a moderate decrease.

## 4. Discussion

In general, job burnout of bus drivers tended to decrease over time, of which the most considerable component was the decrease in reduced personal accomplishment. The second component was cynicism, and the third was emotional exhaustion. It showed that the driver’s burnout was mainly reflected in personal accomplishment, suggesting that it was effective to enhance personal accomplishment to reduce driver burnout. These findings were consistent with the observation in previous studies in which the professional efficacy dimension was a more central symptom in burnout syndrome [35]. In the Introduction, we mentioned the sequential relationship between the development of the three dimensions of MBI, in which exhaustion arose first, followed by cynicism, and the last to appear was reduced personal accomplishment [5]. In this study, the highest average score appeared at reduced personal accomplishment, which might indicate that the participants in this survey, bus drivers, had job burnout for a longer period of time, to the point of low achievement. Therefore, a sense of personal accomplishment might be an important factor in drivers’ burnout experience and company managers of public transportation could alleviate driver burnout by increasing bus drivers’ sense of accomplishment. On the other hand, the Guangdong Statistical Yearbook showed that the number of bus passengers in 2019–2021 was: 2240.9, 1360.66, and 1347.87 million passengers/year, respectively [36]. Was there a correlation between the decline in passengers and the decrease in job burnout of bus drivers? This needs to be explored by further research.

Based on the findings from this research, bus drivers were likely to observe five patterns of job burnout: (1) the moderate-decreased group, (2) the low-stable group, (3) the rapid-decreased group, (4) the moderate-increased group, and (5) the high-stable group. The largest group (39%, class 2) had stable-low levels of job burnout and changed very little over the 26 months. The second group was class 5, which had stable-high levels of job burnout over the 26 months. Compared with class 2, class 5 tended to be stable-high while class 2 was more likely to be stable-low, although both class 5 and 3 had similar membership (25% and 23%, respectively). Class 5 had stable-high levels of job burnout over the 26 months, and class 3 tended to be decreased moderately-. Compared with class 1, class 3 (1%) showed a rapid decrease. It was undeniable that “rapid-decreased” was very researchable for reducing driver’s burnout. However, in the regression, the results of the regression might be biased if compare “rapid-decreased” with other groups, because the number of “rapid decrease” (*n* = 141) was relatively small while “high-stable” (*n* = 3216), “low-stable” (*n* = 5062). We would like to interview “rapid-decreased” as a next step. This finding indicated that most bus drivers displayed a stable low in job burnout. Our data were similar to the result from Benjamin B.D’s study in which burnout was relatively stable for employees without company changes [8]. However, alternatively, it is possible that it was because they had low and stable job burnout that they stayed in the position, while bus drivers with high burnout had left and were not included in our study for this longitudinal survey.

Our key findings were that daily physical exercise, depression symptoms, anxiety symptoms, and insomnia were significant predictors. Specifically, the odds of membership in the job burnout group (versus those higher job burnout classes or the class increasing in job burnout) were lower or more stable for respondents who did daily physical exercise for more than 30 min. This was consistent with a systematic review and meta-analysis showing physical activity was beneficial for burnout syndromes [37,38] and vigorous physical exercise buffers the detrimental effects of job demands on burnout symptoms [39]. As for managers, promoting wellness through workplace physical activity policies, incentives, and support had the potential to prevent burnout [40]. Physical exercise and other enjoyable activities outside of work could promote recovery and reduce resource loss [41,42]. Drivers can perform micro-activities during breaks, which can effectively reduce fatigue and increase vitality, especially for drivers who rest less frequently, the effect of this recovery will be more obvious, such as stretching activities, breathing fresh air outside, or facial muscle relaxation [11].

Job burnout was associated with mental health. In our study, depression, anxiety, and insomnia were important predictors of driver burnout. This indicates that mentally healthier people were more resilient to occupational stress and less likely to suffer burnout. Similarly, a meta-analysis showed a significant association between burnout and anxiety and depression [24]. In a memory test by Bianchi, R, burnout and depression were found to be associated with the decreased recall of positive items and increased recall of negative items. This might explain why drivers with more depressive symptoms were more likely to feel burnout [25]. Furthermore, many studies have shown that the relationship between depression and burnout is bidirectional, i.e., they interact in a vicious cycle or spiral downward, with burnout levels predicting increases in depression over time and depression levels predicting increases in burnout over time [42,43]. This is also worth noting in future study. Insomnia was statistically significantly correlated with job burnout [44]. Insomnia and job burnout interacted with each other: job burnout might indirectly or directly (through chronically elevated cortisol) increase the risk of insomnia, while insomnia might promote burnout [28]. This might contribute to the fact that drivers with more insomnia are more likely to feel burnout. For safety reasons, the bus company had performed regular physical health and mental health examinations on bus drivers. Subjects with significant body disease and mental illness would not be allowed to be on the driving team. In support of this, these two variables were not significantly associated with job burnout in the results of our regression analyses. To reduce burnout, drivers could pay attention to their emotions and seek help when necessary. In addition, company managers could start by improving drivers’ state of mental health, paying attention to drivers’ emotions, following those with sleep problems closely, and increasing the company’s humanistic care.

In addition to the factors above, several demographics and burnout-related factors were also related to changes in job burnout. As for the number of children, if the driver was changing (e.g., moderate-decreased and moderate-increased), burnout was changing, and the slope was steep. Having more children was associated with worse burnout scores. If the driver was in a stable state (e.g., low-stable and high-stable) and the slope of burnout did not change, drivers with more children were more likely to be in a peaceful state in their careers. In combination with age, the older the driver and the more children compared to rapid-decrease and moderate-decreased, the more likely the driver was in a precarious state. Age was unlikely to be a risk factor for low-stable, because the odds ratio (OR) was only 1.01. Low odds ratio meant that the effect of age was minimal and insignificant.

Regarding the variables of drivers’ financial status, financial status was a risk factor for driver burnout, except for drivers who already have been in low burnout. This suggested that company managers could try to increase burnout pay and invite experts to give lectures on financial literacy. Financial literacy was overlooked as a solution for burnout. Improving financial literacy was the first step in helping tackle financial problems that might worsen burnout [45]. On the variable of daily working hours, if the status changes (e.g., moderate-decreased and moderate-increased), the longer the hours worked, the worse the burnout. This may have an interaction effect, and this is worth continuing to discuss.

Moreover, although it has been reported that psychophysiological disorders were positive predictors of exhaustion and cynicism [4], this is not the case in our study. Drivers are responsible for the safety of passengers in the city. Our study cared about the psychological health of them and explored the long-term development of job burnout, playing an active role in caring for bus drivers and bus companies caring about the mental health of bus drivers. This study paid attention to long-term job burnout of bus drivers and showed that most drivers had low levels of burnout and there were drivers whose job burnout increased or decreased over time. This has implications for subsequent research into the factors influencing job burnout of bus drivers. In this study, we made a preliminary exploration of the trends in job burnout, but it is not enough. For future research, we want to reach out further to the driver community and conduct interviews to understand the factors that influence job burnout.

This article had the following limitations. Variables such as number of children, financial status, and driving years were discussed, but in terms of the comprehensiveness of the variables in JD-R, our study did not cover the variables involved in those theories well enough to argue for it, which is a shortcoming of our article. Regarding the JD-R, the individual’s resources might include family functioning, social support, emotion regulation, etc., but in this study, none of these variables were measured. Furthermore, we focused more on negative variables and we covered fewer positive variables such as social support or quality of leadership. Factors related to driver personality and job characteristics have predictive effects on burnout, but this part of the study lacked exploration. Moreover, burnout, depression symptoms, anxiety symptoms and insomnia were examined through self-reported questionnaires; thus, participants’ answers could be affected by potential self-report biases. In addition, we did not control for confounding factors between measurements, such as the COVID-19 epidemic, the gradual boom of the subway resulting in lower bus trips, bus accidents in the news during this period, etc. This study also ignored group heterogeneity, such as whether they worked night shifts, different driving routes, etc.

## 5. Conclusions

In the 26-month longitudinal research, we found evidence that bus drivers’ burnout showed an overall downward trend, mainly reflected in personal accomplishment. Public transportation companies could make certain interventions in this area to improve drivers’ sense of accomplishment in a targeted manner, thus alleviating burnout. Mentally healthier drivers and those who were usually exercising were more resilient to occupational stress and less likely to suffer burnout. These results have practical implications. Public transportation companies could intervene to alleviate burnout by addressing the above factors. Future research could select subjects from the group for targeted interviews.

## Figures and Tables

**Table 1:** Basic descriptive statistics of participants (*n* = 12,793).

Characteristics	M ± SD/*n*(%)
Age	47.27 ± 6.48
Gender	
Female	179 (1.40)
Male	12,614 (98.60)
Marital status	
Unmarried	1394 (10.90)
Married	11,399 (89.10)
Number of children	1.49 ± 0.80
Financial situation	
Higher or equal to normal	6787 (53.05)
Lower to normal	6006 (46.95)
Body disease	
Yes	223 (1.74)
No	12,570 (98.26)
Mental illness	
Yes	18 (0.14)
No	12,775 (99.86)
Driving years	12.39 ± 7.01
Work hours	
Less than 8 h	5442 (42.54)
More than 8 h	7351 (57.46)
Daily Physical exercise	
Less than 30 min	7175 (56.09)
More than 30 min	5618 (43.91)
Depression symptoms	
No	10,116 (79.07)
Yes	2677 (20.93)
Anxiety symptoms	
No	10,985 (85.87)
Yes	1808 (14.13)
Insomnia	
No	10,870 (84.97)
Yes	1923 (15.03)
Aggression	39.29 (14.18)
Job burnout	
T1	21.59 ± 15.98
T2	19.81 ± 15.81
T3	17.79 ± 15.82

**Table 2:** Relative fit indices for 1 through 6 class models.

Model	AIC	BIC	Δ BIC	Class size	Entropy	LMRT	BLRT	Par.
1 Class	316,256	316,315		100%	-	-	-	8
2 Class	312,114	312,196	−4119	63%, 37%	0.909	*p* < 0.001	*p* < 0.001	11
3 Class	310,847	310,951	−8755	61%, 37%, 1%	0.946	*p* < 0.001	*p* < 0.001	14
4 Class	309,511	309,637	−1314	53%, 30%, 2%, 15%	0.920	*p* < 0.001	*p* < 0.001	17
5 Class	308,689	308,838	−799	23%, 39%, 1%, 12%,25%	0.909	*p* < 0.001	*p* < 0.001	20
6 Class	307,886	308,057	−781	38%, 1%, 12%, 1%, 25%, 23%	0.918	*p* = 0.088	*p* < 0.001	23

BIC = Bayesian information criterion; AIC = Akaike information criterion; Δ BIC = change in Bayesian information criterion; LMRT = Lo–Mendel–Rubin likelihood ratio test; LRT = Bootstrap likelihood ratio test; Par. = parameters in the model.

**Table 3:** Average Latent Class Probabilities for Most Likely Latent Class Membership (Row) by Latent Class (Column).

	1(%)	2(%)	3(%)	4(%)	5(%)
1	93.2	3.9	0.5	0.0	2.5
2	2.9	95.1	0.0	1.3	0.7
3	7.8	0.0	90.0	0.0	2.2
4	0.0	3.3	0.0	92.3	4.4
5	2.2	1.0	0.1	2.2	94.6

**Table 4:** Predictors (odds ratios) of job burnout.

	“Moderate-Increased” vs. “Low-Stable”	“Moderate-Decreased” vs. “High-Stable”	“Low-Stable” vs. “High-Stable”	“Moderate-Increased” vs. “Moderate-Decreased”
Age	1.01 (1.00,1.02) *	1.00 (0.99,1.01)	0.99 (0.98,1.00)	1.02 (1.00,1.03) *
Gender (female = reference)				
Male	1.15 (0.66,2.03)	0.99 (0.60,1.61)	1.21 (0.81,1.82)	0.94 (0.51,1.73)
Marital status (else = reference)				
Married	0.97 (0.77,1.21)	1.03 (0.85,1.25)	1.03 (0.87,1.22)	0.97 (0.76,1.23)
Number of children				
	1.03 (0.96,1.10)	0.96 (0.88,1.05)	0.86 (0.80,0.92) ***	1.15 (1.04,1.27) *
Financial situation (higher or equal to regular = reference)				
Lower to normal	1.15 (1.00,1.31)	1.26 (1.12,1.42) **	1.19 (1.07,1.32) *	1.21 (1.05,1.40) *
Body disease (No = reference)				
Yes	1.23 (0.75,2.02)	1.13 (0.71,1.81)	1.12 (0.74,1.71)	1.24 (0.73,2.09)
Mental illness (No = reference)				
Yes	4.46 (0.12,170.23)	0.93 (0.14,6.04)	3.50 (0.12,104.86)	1.19 (0.15,9.33)
Driving years				
	1.02 (1.01,1.03) **	1.00 (1.00,1.01)	1.03 (1.02,1.03) ***	0.99 (0.98,1.01)
Daily work hours (less than 8 h = reference)				
More than 8 h	1.08 (0.94,1.23)	1.13 (1.00,1.27)	0.92 (0.83,1.02)	1.33 (1.15,1.53) *
Daily physical exercise (less than 30 min = reference) †				
More than 30 min	0.72 (0.63,0.82) ***	0.69 (0.61,0.78) ***	0.57 (0.51,0.63) ***	0.87 (0.76,1.00) *
Depression symptoms (no = reference)				
Yes	2.51 (1.99,3.17) ***	2.55 (2.06,3.15) ***	3.04 (2.53,3.65) ***	2.10 (1.64,2.70) ***
Anxiety symptoms (no = reference)				
Yes	2.19 (1.65,2.92) ***	2.42 (1.80,3.25) ***	2.17 (1.71,2.76) ***	2.44 (1.76,3.37) ***
Insomnia (no = reference)				
Yes	2.27 (1.80,2.84) ***	2.06 (1.63,2.60) ***	1.90 (1.56,2.31) ***	2.46 (1.90,3.17) ***
Aggression				
	1.00 (0.99,1.01)	1.01 (1.00,1.01) *	1.02 (1.02,1.02) ***	0.99 (0.98,0.99) ***

Note: * *p* < 0.05, ** *p* < 0.01, *** *p* < 0.001, † was collected at T3, all other variables were collected at T1.

## Data Availability

The database is not available for direct access, but can be requested from researchers.

## Supplementary Materials

The following supporting information can be downloaded at: https://www.mdpi.com/article/10.3390/ijerph192417098/s1, File S1: Introduce about Sojump; File S2: The translated version of demographic part of questionnaire; File S3: The GMM code in mlpus. Ref. [46] is cited in Supplementary Materials.
