# Supplementary material for: Trajectories of Job Burnout among Bus Drivers in China: A Three-Year Follow-Up Study
Source: Int J Environ Res Public Health. 2022 Dec 19;19(24):17098. doi: 10.3390/ijerph192417098 (PMC9779569; doi:10.3390/ijerph192417098)
Supplement: Supplementary file 1 [file ijerph-19-17098-s001.zip › ijerph-1989026-supplementary.pdf]

File S1. Introduce about Sojump

Sojump（www.sojump.com） is a professional online platform. The functions include online questionnaire, examination, assessment, voting. Focused on providing users with powerful, user-friendly services, including online design questionnaires, data collection, custom reports, survey results analysis. Compared with traditional survey methods, sojump has the obvious advantages of fast, easy to use, low-cost, has been widely used by a large number of enterprises and individuals.

File S2. The translated version of demographic part of questionnaire

Dear driver:

shalom! In order to understand your mental health status, improve your mental health level, now on your psychological characteristics and tendency to investigate, to provide a scientific basis for the follow-up psychological adjustment, please be sure to fill in truthfully, thank you!

1. Base situation

Guidance: Please answer the following questions truthfully according to your actual situation.

Name: ________________

Age: _________________

Mobile phone number: ________________

ID Number: ________________

Employee card number: _____________

1. Gender: [Single choice] *

○ man

○ woman

2. Education [Single choice] *

○Graduate from elementary school

○Graduation from junior high school

○High school graduation

○University graduation

○ Other __________________ *

3. Marital status: [Single-choice] *

○Married

○Unmarried

○Divorced

○Widowed

4. Number of your children: _______ [blank] *

5. How would you rate your family's financial situation? [Single choice] *

○Significantly higher than normal

○Somewhat higher than normal

○Normal level
○Somewhat below normal
○Significantly below normal

6. Are you suffering from severe physical disease? [Single choice] *
○A. No
○B. Yes (please explain): ________________ *

7. Have you ever been diagnosed with a mental illness? [Single choice] *
○A. No
○B. Yes (please explain): ________________ *

8. Are you used to taking sleeping pills to help you get sleep [Single choice] *
○A. No (please skip to question # 13)
○B. Yes (please specify the name of the drug you have taken): ________________ *

9. The number of times you take sleep-aid medication per week is [single-choice] *
○Two or more times per day
○1 time per day
○Once every 2 days
○2 times a week
○1 time per week or less

10 The dose you take is [single choice] *
○Half a capsule or less each time
○1 capsule each time
○2 capsules each time
○3 or more capsules each time

11 How long you keep taking the sleep-aid medication [single-choice] *
○Lasting for 1 month or less
○Lasting for 2 months
○Lasting for 3 months
○Lasting 3 months to 6 months
○Lasts six months or longer

12. How many years have you been in the business? [fill in the blank question] *

____

13. How many hours do you work per day? [single choice]*
○8 hours or less
○9-10 hours
○11-12 hours
○13-14 hours

○15 hours and above

14 In the past two weeks, what was the average amount of time spent exercising daily? [Single choice] *
○A. Never
○B. Less than 30 minutes
○C. For about 30-60 minutes
○D. More than 60 minutes

File S3. The GMM code in mlpus

S3.1 One group GMM model in Mplus

```
TITLE:    GMM FOR ONE GROUP
DATA:
  FILE IS D: BB.dat;
VARIABLE:
  NAMES ARE ID A1 A2 A3 A4 A5 A6 A7 A8 A9 A10 A11 A12 A13 A14
A15
  A16 PHQ1 GAD1 ISI1 AQ1 MBI1 PHQ2 GAD2 ISI2 MBI2 PHQ3
  GAD3 ISI3 MBI3;
  usevariables are    MBI1    MBI2    MBI3;
  CLASSES = c (1);
  auxiliary=ID A2-A16;
ANALYSIS: TYPE = MIXTURE;
  starts=800 20;
  processor=4;
MODEL:
  %OVERALL%
  i s | MBI1@0 MBI2@7 MBI3@13;
OUTPUT:   TECH1 TECH8 TECH11 TECH14;
savedata: FILE= C5.CSV;save=cprob;
plot:
        type is plot3;
```

S3.2 Five groups GMM model in Mplus

The only difference between one group and the other group is START, we set START 800 10 at first, and find the best loglikelihood value has been replicated, then we change START to 800 20 to make sure the best loglikelihood value stays the same1. We use the same way for class 2 through class 6. The syntax below was for class 5. We did not set output in this stage because BLRT can be boosted by adding "optseed" of the best loglikelihood value5, so we estimated BLRT and LMRT in the next stage.

```
TITLE:    GMM FOR FIVE GROUP
DATA:
```

```
   FILE IS D: BB.dat;
VARIABLE:
   NAMES ARE ID A1 A2 A3 A4 A5 A6 A7 A8 A9 A10 A11 A12 A13 A14
A15
   A16 PHQ1 GAD1 ISI1 AQ1 MBI1 PHQ2 GAD2 ISI2 MBI2 PHQ3
   GAD3 ISI3 MBI3;
   usevariables are     MBI1     MBI2     MBI3;
   CLASSES = c (5);
   auxiliary=ID A2-A16;
ANALYSIS: TYPE = MIXTURE;
   starts=800 20;
   processor=4;
MODEL:
   %OVERALL%
   i s | MBI1@0 MBI2@7 MBI3@13;
OUTPUT:   TECH1 TECH8 TECH11 TECH14;
savedata: FILE= C5.CSV;save=cprob;
plot:
          type is plot3;
          series=MBI1 MBI2 MBI3 (*);
         series=MBI1 MBI2 MBI3 (*);
```

S3.3 Multinomial logistic regression

According to Asparouhov, T.; Muthén, B.[1], multinomial logistic regression was estimated based on logits for the classification probabilities for the most likely latent class membership by previous stage's output. The output command tech11 will give results for different reference groups; CINTERVAL will estimate 95% CI.

```
TITLE:   Multinomial logistic regression
DATA:
   FILE IS C5.CSV;
VARIABLE:
   NAMES ARE   MBI1-MBI3 ID A2-A11 A14-A17 P1-P9 N;
   usevariables are A2-A11 A14-A17 N;
   CLASSES = c(5);
   NOMINAL=N;
ANALYSIS: TYPE = MIXTURE;
   starts=0;
   processor=4;
MODEL:
MODEL: %OVERALL%
          C ON A2-A11 A14-A17;
```

```
%c#1%
[n#1@3.641];
[n#2@0.736];
[n#3@-1.855];
[n#4@-8.430];
%c#2%
[n#1@1.206];
[n#2@4.977];
[n#3@-8.800];
[n#4@0.393];
%c#3%
[n#1@2.041];
[n#2@-9.441];
[n#3@4.259];
[n#4@-9.441];
%c#4%
[n#1@-8.628];
[n#2@-0.081];
[n#3@-10.734];
[n#4@2.989];
%c#5%
[n#1@-3.760];
[n#2@-4.477];
[n#3@-6.869];
[n#4@-3.829];
output:
tech11; CINTERVAL;
```


1. Asparouhov, T.; Muthen, B. Auxiliary Variables in Mixture Modeling: Three-Step Approaches Using Mplus. *Structural Equation Modeling-a Multidisciplinary Journal* **2014**, *21*, 329-341, doi:10.1080/10705511.2014.915181.
